# NRDTD: a database for clinically or experimentally supported non-coding RNAs and drug targets associations

**DOI:** 10.1093/database/bax057

**Published:** 2017-07-21

**Authors:** Xing Chen, Ya-Zhou Sun, De-Hong Zhang, Jian-Qiang Li, Gui-Ying Yan, Ji-Yong An, Zhu-Hong You

**Affiliations:** 1 School of Information and Control Engineering, China University of Mining and Technology, Xuzhou 221116, China; 2 College of Computer Science and Software Engineering, Shenzhen University, Shenzhen 518060, China; 3 Academy of Mathematics and Systems Science, Chinese Academy of Sciences, Beijing 100190, China; 4 School of Computer Science and Technology, China University of Mining and Technology, Xuzhou 21116, China and; 5 Xinjiang Technical Institute of Physics and Chemistry, Chinese Academy of Science, Urumqi 830011, China

## Abstract

In recent years, more and more non-coding RNAs (ncRNAs) have been identified and increasing evidences have shown that ncRNAs may affect gene expression and disease progression, making them a new class of targets for drug discovery. It thus becomes important to understand the relationship between ncRNAs and drug targets. For this purpose, an ncRNAs and drug targets association database would be extremely beneficial. Here, we developed ncRNA Drug Targets Database (NRDTD) that collected 165 entries of clinically or experimentally supported ncRNAs as drug targets, including 97 ncRNAs and 96 drugs. Moreover, we annotated ncRNA-drug target associations with drug information from KEGG, PubChem, DrugBank, CTD or Wikipedia, GenBank sequence links, OMIM disease ID, pathway and function annotation for ncRNAs, detailed description of associations between ncRNAs and diseases from HMDD or LncRNADisease and the publication PubMed ID. Additionally, we provided users a link to submit novel disease-ncRNA-drug associations and corresponding supporting evidences into the database. We hope NRDTD will be a useful resource for investigating the roles of ncRNAs in drug target identification, drug discovery and disease treatment.

**Database URL:** http://chengroup.cumt.edu.cn/NRDTD

## Introduction

Human genome undergoes transcription to generate thousands of RNA molecules. ENCODE consortium revealed that about 70% of the genome is transcribed for RNA molecules that have no proteins coding capacity (1). They are known as non-coding RNAs (ncRNAs) including circular RNAs (circRNAs) (2), extracellular RNAs (3), intronic RNA (4), long non-coding RNAs (lncRNAs) (5), microRNAs (miRNAs) (6) and Piwi-interacting RNAs (piRNAs) (7). Although ncRNAs lack the potential to encode proteins, they play important roles in cellular functions, and their deregulation heavily contributes to various pathological conditions (8). The capacity of ncRNAs to affect gene expression makes them potential targets for drug development (9).

Emerging classes of ncRNA targets include miRNAs (10), intronic RNA (4) repetitive RNAs (11) and lncRNAs (1, 12, 13) which perform different functions *in vivo*, providing a variety of opportunities and challenges for drug discovery. MiRNAs form a major class of functional ncRNAs (14, 15). Both miRNA inhibitors and mimics can regulate its activity, so they are currently developed against a variety of targets. For example, mimics of miR-34 can repress oncogene expression and block tumor growth (16); single-stranded oligonucleotides complementary to miR-122 are being applied to treat Hepatitis C virus (17) and oligonucleotides complementary to miR-21 are being developed to treat Alport nephropathy (18). Intronic RNA is one of the most prevalent species of ncRNA as approximately 30% of the genome encodes it (4). Targeting key splicing control sequences within introns can lead to the biosynthesis of different protein isoforms and offer opportunities for therapeutic development (19). Nusinersen targeting intron 7 within Survival of Motor Neuron 2 (SMN2) restored splicing and facilitated production of full-length SMN2 in spinal muscular atrophy (SMA) (20). Phase I clinical trials of nusinersen have been completed in patients with SMA (21) and further trials are ongoing. >40 neurological diseases are caused by repetitive sequences within DNA (22, 23). Repetitive RNA and nearby sequences within introns are attractive targets. A research group developed small molecules hybrids to target CUG repeats to silence nuclear DMPK transcripts with high selectivity, which effectively reduce the myotonic dystrophy type 1 symptoms in mice (24, 25). A phase I/II clinical study was initiated in 2014 to evaluate its safety and tolerability (https://www.clinicaltrials.gov/ct2/show/NCT02312011). LncRNAs are a diverse group of transcripts whose natural functions and potential as drug targets remain largely undefined. Several lncRNAs that have been the focus of studies are novel targets for the discovery and development of agents. For example, MALAT1, HOTAIR and NORAD (26–28) in cancer cells can elicit effective cancer inhibitory effect. These lncRNAs linked to cancer may present potential therapeutic targets. Based on these experimental or clinical data, the therapeutic approaches targeting ncRNAs in treating human disease are gaining enormous momentum.

To date, several ncRNA-related databases have been developed. For example, NONCODE is an integrated knowledge database including almost all ncRNA classes (29). NRED is a database for lncRNA expression data (30). HMDD is a database for experimentally miRNA-disease associations (31). LncRNADisease provide experimentally lncRNA-disease associations and predicted results (32). These databases have shown their great help in providing valuable ncRNA-related information (33).

NcRNAs are a promising class of targets for drug development (34, 35). Nowadays, there are some drug-target databases such as DrugBank (36), Therapeutic target database (TTD) (37) and SuperTarget (38). However, to our knowledge, there is no special database providing comprehensive resources for ncRNAs acting as drug targets. To design effective drugs for clinical treatment, there is a pressing need to improve our understanding of relationship between ncRNAs and drugs and their association mechanisms. To fill this gap, we developed a literature-based ncRNA Drug Targets Database (NRDTD), which was proposed to assist with investigating the relationship between ncRNAs and drugs and facilitate the researches on ncRNA-based therapies.

## Materials and methods

To collect the clinically or experimentally supported ncRNA and drug target associations, we firstly performed an extensive literature query of Pubmed database using a list of keywords, such as ‘non-coding RNA’, ‘miRNA’, ‘lncRNA’, ‘piRNA’ and ‘drug targets’ on August 15, 2016. There are >5000 abstracts when searching ‘non-coding RNA’ and ‘drug targets’ (PubMed query strings (‘non coding rna’[All Fields] AND ‘drug targets’[All Fields])), >2000 abstracts when searching ‘miRNA and drug targets’ (PubMed query strings (‘microrna’ [All Fields] OR ‘mirna’[All Fields]) AND ‘drug targets’[All Fields])), >100 abstracts when searching ‘lncRNA and drug targets’ (PubMed query strings (‘long noncoding rna’[All Fields] OR ‘lncrna’[All Fields]) AND ‘drug targets’[All Fields])) and >3000 abstracts when searching ‘piRNA and drug targets’ (PubMed query strings (‘pirna’[All Fields]) AND (‘drug targets’[All Fields])), respectively. Through primary screening, we deleted those references which are repeated and clearly irrelevant to associations between non-coding RNA and drug targets and reserved >3000 abstracts. Then we manually retrieved entries related to ncRNAs and drug targets associations by reading abstracts. Only entries with clear clinical or experimental evidences were chosen. The information for these chosen entries was extracted from the full text articles. Furthermore, since many ncRNAs can have an indirect effect on a particular drug, we chose only the entries for which there was a direct interaction. That means the drugs directly act on these ncRNAs to regulate their expression or function without intermediate regulator. It may include physical interaction or chemical modification and other direct regulation mechanisms. Hyperlinks to the original articles in PubMed database were also provided (39).

**Figure 1.**
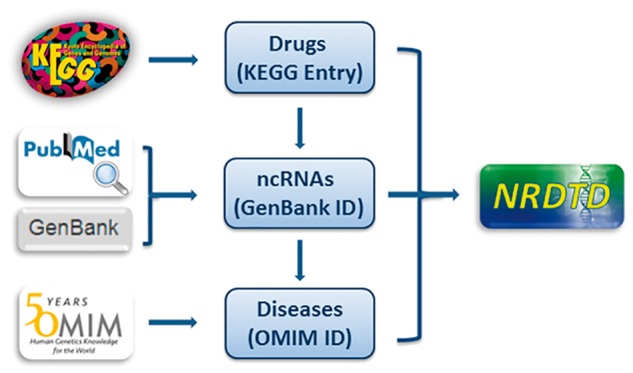
The ﬂowchart of NRDTD construction. The ﬂowchart shows the process of data processing and information integration.

Every entry contains four major items, which are drug name, targeting ncRNA name, disease name and the publication PubMed ID. We further annotated the drug, ncRNA sequence and disease information with links to KEGG (40), Genbank (41) and OMIM database (42), respectively (Figure 1). To give more information about drugs, we added description for drugs from KEGG. As many drugs such as traditional Chinese medicine extracts and newly discovered compounds don’t have corresponding KEGG links, we also added description for them from PubChem, Wikipedia or the corresponding references to make sure that each drug has necessary information. Considering NRDTD is a database taking the drugs as main part, perfect information about drugs is essential. Thus we also provided cross-links to DrugBank (36), PubChem (43) and CTD database (44) for users to get extra information about drugs easily. Moreover, we provided detailed descriptions for the associations of ncRNAs and drug targets for each entry. In addition, ncRNAs were grouped by type. Taking into account that the number of ncRNAs as drug targets is really small, more information about disease states and ncRNA biochemical pathways were included in the database. We provided the pathways and functions annotation for ncRNAs from the miRBase (45) and LncRNAWiki (46) databases. The links in these two databases also can be accessed directly in the NRDTD. In addition, we provided the description of the corresponding entries from HMDD (31) and LncRNADisease (32) databases to help users to understand the association among ncRNAs, diseases and drugs.

In the NRDTD database, all data were organized in our web server using the browser/server framework based on PHP, Apache2 and MySQL system (47). The database is available at http://chengroup.cumt.edu.cn/NRDTD.

## Results and discussion

**Figure 2.**
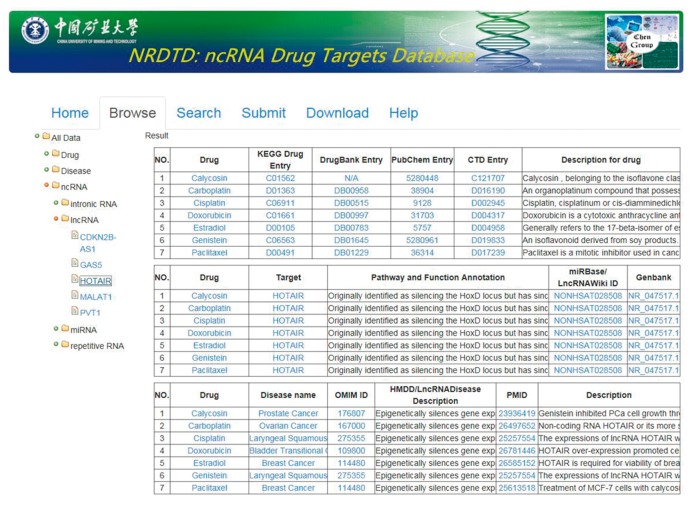
The NRDTD user interface showing the browse page.

In the current version, NRDTD collects 165 entries that include 97 ncRNAs and 96 drugs from >3000 papers. The data in NRDTD can be easily accessed in various ways. First, users can browse the NRDTD by ncRNA names or drug names. When clicking on one ncRNA, drug or disease in the ‘Browse’ page, NRDTD returns the corresponding list of entries (Figure 2). For example, to browse the entries related to the candidate ncRNAs, please click ‘ncRNA’ and select the one that users are interested in. The result will be shown in the right panel. To browse the entries related to the candidate drug or disease, please click ‘drug’ or ‘disease’ and select the one that users are interested in. If the users want to read more information about the pathways and functions annotation or description, they can double click corresponding content to get complete information. Additionally, for the users to easily explore all the associations among ncRNAs, drugs and diseases, we added internal links of them. For example, when the users look at Browse > All data > ncRNA > miRNA > let-7, if they want to explore if that given drug ‘Genistein’ has effects on a different miRNA as well, they can click the drug name and all the entries related to‘Genistein’ will be shown in the right panel. Similarly, they can click the ncRNA or disease names directly to look at all related entries. Second, we provided ‘search’ functions for the entries by the full or partial names of ncRNAs or drug in the ‘Search’ page. The ‘Search’ is case-insensitive. Moreover, all data in the database, including disease-related ncRNA–drug associations, descriptions of associations, publication PubMed ID, all ncRNA names, drug names and disease names, can be downloaded.

Aside from data retrieval from NRDTD, users can also submit novel data into the database. They may first search NRDTD to check whether their data have already been deposited into the database. If not, they can upload the related information. The novel entries will be forwarded to the NRDTD developers via email and will become available after a manual check and confirmation. In addition, a detailed tutorial for the usage of the database is available in the ‘Help’ page.

**Figure 3.**
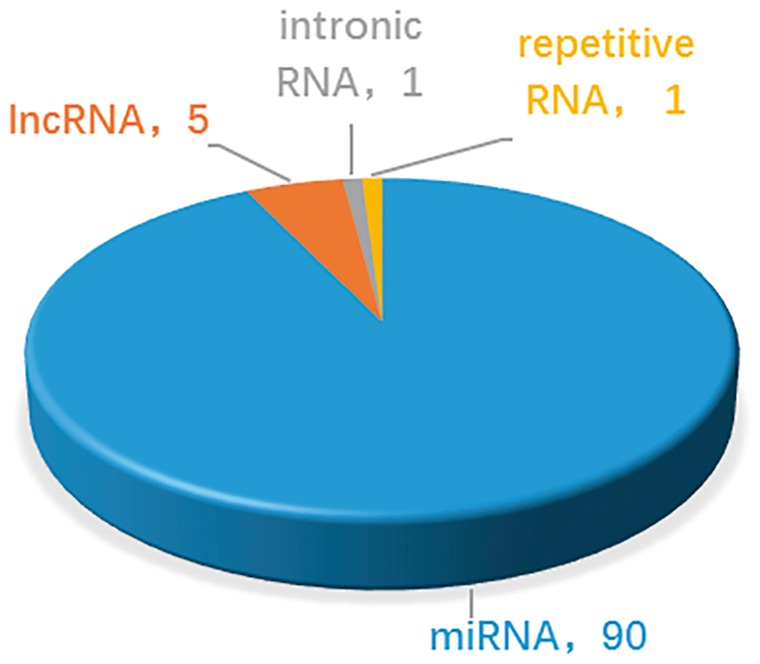
Statistics and distribution of different types of ncRNAs as drug targets in the NRDTD database.

At present, the number of ncRNAs as drug targets is not very large. This is partly due to the discovery process, which is time-consuming. On the other hand, uncertainty about how ncRNAs function makes identification even more challenging. Thus the common feature of ncRNAs which are targetable by drugs is that their functions and mechanisms are well-studied and clear. These ncRNAs, such as let-7, miR-21, MALAT1, were well known about their function and mechanisms. On the contrary, ncRNAs without specific biological functions are difficult to become drug targets. As shown in Figure 3, in the near term, compounds that targeting miRNAs act through the best under stood mechanisms and will be the focus of most clinical development. In the longer term, understanding of the mechanism of lncRNAs and other ncRNAs may grow. In addition, new classes of potential non-coding targets may emerge, such as piRNAs and circRNAs, which have been reported with more and more important biological functions. The important roles of ncRNAs in drug discovery are attracting more scientific interest (24, 48, 49). As our understanding of mechanisms of ncRNAs improve, the design of effective drug development will gain a firmer foundation and the likelihood of clinical success will increase. Therefore, more ncRNAs and drug targets associations are expected to be reported and integrated into NRDTD. The purpose of NRDTD is to provide comprehensive resource about associations among ncRNAs, drugs and disease. Along with the number of associations in NRDTD increase consistently, NRDTD will become a high-quality database for prediction of associations among ncRNAs, drugs and disease with perfect functions finally and make bigger contribution to solve actual biological problems. For example, as the drug-resistant problem become more and more serious, it is important to discover new uses of old drugs and develop synergistic drug combination. If we want to develop synergistic drug combination strategy for gastric cancer, we can look at the related drugs in NRDTD including cisplatin, cinobufacin, isoproterenol, diallyl disulfide, rosmarinie acid and so on. Then we can try to combine them to enhance the anti-cancer efficiency. More importantly, we can search the ncRNAs related to gastric cancer and look at the drugs targeting them. For instance, via the gastric cancer related ncRNA miR-155, we find TanshinoneIIA also targets miR-155 as rosmarinie acid. Although there are not directly associations between TanshinoneIIA, we can try to test its efficiency to anti gastric cancer as they have the same targets. Furtherly, we may add it to the synergistic drug combination strategy. The NRDTD represents the first step in our ncRNA-based drug targets project. In the future, further extensions will be developed. The NRDTD will be updated continually and computational methods would be developed to predict novel ncRNAs and drug targets associations.

## Conclusion and future direction

Increasing studies have shown that ncRNAs have important functions and are involved in drug discovery. Targeting ncRNA has the potential to offer novel therapeutic opportunities. Given the significant effect brought out by targeting ncRNAs, it is foreseeable that research on ncRNA-based therapies will gain greater interest in the future. In this article, we present the NRDTD which is the first database focusing on ncRNA-based drug targets. It integrates various types of data related to ncRNAs and drug targets associations. We plan to update NRDTD every 2 months with the experimentally supported disease-related ncRNA-drug association data from newly published references. Meanwhile, some new tools for analysing ncRNA-drug association data is being developed and will be integrated into the NRDTD database in the future. For example, we will develop interacting partner-based methods to predict novel disease-related ncRNA-drug association and expect to integrated these methods into database in the near future. We believe that NRDTD would be useful for the studies of ncRNAs and drug targets, and will provide more help when it will integrate more data and tools in the future.

## Availability

NRDTD database is freely available at http://chengroup.cumt.edu.cn/NRDTD.
